# Use of cover data to model species abundance distributions through continuous probability functions

**DOI:** 10.1038/s41598-025-94587-w

**Published:** 2025-04-02

**Authors:** Halime Moradi, Paulo Inácio Prado, Jens Oldeland, Paulo A. V. Borges, Simone Fattorini

**Affiliations:** 1 CEN Center for Earth System Research and Sustainability, Institute of Geography, University of Hamburg, Bundesstrasse 55, 20146 Hamburg, Germany; 2 Biosciences Institute at São Paulo University, Rua do Matão, travessa 14, nº 321, São Paulo, 05508-900 SP Brazil; 3 Institute for Globally Distributed Open Research and Education (IGDORE), Burgunderweg 9d, 22453 Hamburg, Germany; 4 University of Azores, Centre for Ecology, Evolution and Environmental Changes (cE3c)/Azorean Biodiversity Group, CHANGE—Global Change and Sustainability Institute, Faculty of Agricultural Sciences and Environment, Rua Capitão João d´Avila, Pico da Urze, Angra do Heroísmo, 9700-042 Portugal; 5 Department of Life, Health and Environmental Sciences, University of L’Aquila, Via Vetoio, L’Aquila, 67100 Italy

**Keywords:** Alborz mountains, Community ecology, Continuous distributions, Elevational gradient, Plant species abundance distributions, Weibull model, Ecology, Ecology, Environmental sciences

## Abstract

Species abundance distribution (SAD) models describe the abundances of the species within ecological communities. SAD modelling has been developed using frequency distributions that require the use of count data (number of individuals). However, many organisms, such as most plants, cannot be counted. Instead, abundance is estimated using cover values. We show here how SAD approaches conceived for modelling frequency distributions based on counts can be modified to deal with continuous distributions and provide an application using relative plant cover as a continuous measure of abundance. We applied several SAD models using continuous probability functions to investigate how plant SADs changed along a wide (2,500 m) elevational gradient in the Alborz Mountains (Iran). We found that most communities were adequately fitted by the Weibull distribution, whose parameters changed along the gradient in response to the interplay between biotic and environmental filtering processes. The use of continuous probability functions in SAD modelling should be encouraged in research dealing with plant communities and other organisms for which counting individuals is theoretically or practically impossible.

**Supplementary Information:**

The online version contains supplementary material available at 10.1038/s41598-025-94587-w.

## Introduction

Species abundance distribution (SAD) models describe the abundance of the species within an assemblage^1^. Although most SADs are characterised by the same basic pattern, in which most species are rare and few are common, communities largely differ in the shape of their SADs^2–4^. The study of SAD patterns has been proven to be of paramount importance in theoretical ecology, applied ecology, and biodiversity management^5,6^. Despite SAD modelling has attracted increasing interest in the last years^1,4–6^, research in SADs has been strongly biased towards countable organisms, as number of individuals are used as measures of abundance in SAD models that are based on fitting frequency distributions. Thus, our knowledge on SAD patterns, although involving a huge number of specific studies and comparative analyses, arise substantially from vertebrates (especially birds) and arthropods^7–10^, and, to a lesser extent, from trees^8,9,11–13^. Since for non-tree plant species abundance is typically expressed as percent cover instead of number of individuals, knowledge of SADs for these organisms is much more limited, and mostly based on the use of rank-abundance plots—an approach in which SADs are not modelled using frequency distributions, but species abundances are plotted against rank order, where rank one corresponds to the species with the highest abundance and so on^3,6,14–17^. Although rank-abundance regressions may offer some advantages to test certain SAD models^18^, they have important disadvantages, being less effective in displaying the entire distribution, especially for rare species; also, they may not convey information about the shape of the distribution, as done by fitting a probability distribution to cover data binned in a histogram. Moreover, regression models fitted to rank-abundance plots require additional assumptions about sampling errors, and may perform poorly because of tied values, such as many records of species in the same cover/abundance class.

Focusing on count data represents a serious limit in SAD studies, as for many organisms it is practically impossible, or even theoretically inappropriate, to distinguish or count individuals. These organisms are certainly not an exiguous minority, as they include, for, example, most of shrubby and herbaceous plants, whose abundance is typically expressed in community ecology as percent cover, point quadrat frequency or biomass, instead of number of individuals^7,19^. The impossibility of counting individuals for most of these species derives from the fact that they are modular organisms and there are obvious difficulties in distinguishing individuals when there is variation in size within a species, as is common in seed plants^3,20^, which leads to the use of relative cover as a measure of abundance. The wide use of cover data in plant ecology also relies on its correlation with biomass, and hence with productivity. Biomass is often considered a good approximation of productivity, especially in grassland communities, but its measurement is time-consuming and destructive^15^. Cover data are strongly correlated with plant biomass (often more than with the number of ramets) and are relatively easy to measure, especially through the use of cover classes that allow rapid visual estimates in the field^15,17^.

In this paper, we illustrate how SAD approaches usually applied for modelling frequency distributions based on counts can be easily used to deal with continuous measures of abundance, such as cover data. To illustrate this approach, and its importance to gain better understanding of SAD patterns, we selected, as a case study of particular relevance, the analysis of changes in the SAD shape of herbaceous plant communities along an elevational gradient. Variation in plant SADs of mountain ecosystems remain virtually unexplored (but see^12^ for a notable exception), which appears quite remarkable, because plant communities on mountains show strong variations in response to environmental changes that occur along the elevational gradient, such as soil properties, temperature, precipitation, insulation, winds, etc.^21–24^. It is not a coincidence that one of the earliest comparative analyses on the SADs was conducted along an elevational gradient in mountain plant communities^25^. To this end, we used data from a wide elevational range, extending for 2,500 m above the treeline in the Alborz Mountains (Iran), within the Caucasus global biodiversity hotspot^26^. This allowed us to apply our approach to grassland plants, which are poorly investigated in SADs studies, despite their high species richness and obvious ecological importance^27^.

Specifically, we tested the hypothesis that SADs change their shape along the gradient in response to variations in the influence of abiotic and biotic factors^12,28^. We hypothesise that at the lowest part of the gradient, biotic interactions play a more important role than abiotic factors. However, with increasing elevation and increasingly harsher conditions, communities become more strongly shaped by environmental filtering^29^. At the highest elevations, environmental conditions are so harsh that even the most successful species have low abundances, leading to SADs influenced by the process of niche allocation, i.e. by the selection of a few stress-tolerant species^30,31^.

To the best of our knowledge, this is the first study that uses continuous distributions to fit SADs to abundances in cover classes and to explicitly investigate variations in SADs along a wide elevational gradient.

## Results

**Fig. 1:**
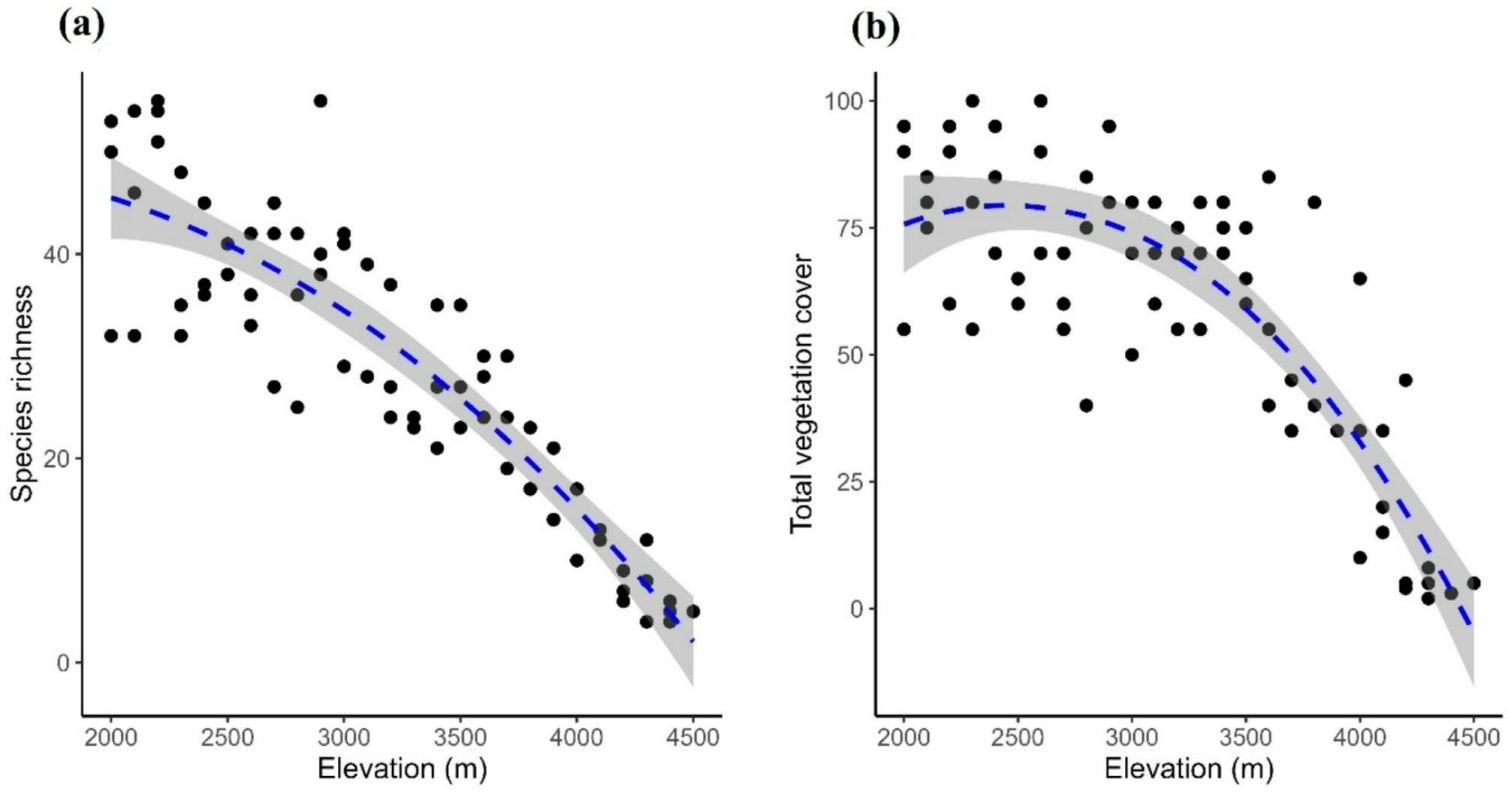
Relationship of (a) species richness and (b) total vegetation cover with altitude along an elevational gradient in the Alborz Mountains (Iran). Vegetation sampling was conducted in 10 m × 10 m square plots. The black points represent observed data, while the blue dashed lines represent the loess smoother. The grey areas show the confidence interval around the fitted line.

A total of 290 plant species were found along the elevational gradient. Plant species richness decreased monotonically (albeit not linearly) with elevation (Fig. 1). On average, 46.3 species were found per 100 m^2^ vegetation at the beginning of the transect at 2,000 m, while only five occurred at the end of the transect. In the middle of the transect, at 3,200 m, an average of 30 species per plot was estimated by the smoothing function. Similarly, the overall vegetation cover decreased with elevation from 80% to less than 5%, following a comparable trend (Table 1; Fig. 1).

**Table 1:** Number of times each species abundance distribution (SAD) model was selected among the equally best supported models (AICc ≤ 2) for the species abundance distributions of vascular plants along an elevational gradient in the Alborz Mountains (Iran). Values of mean species richness and total vegetation cover are also shown.

Elevation (m)	Exponential	Gamma	Lognormal	Pareto	Weibull	Mean of species richness (min-max)	Mean of total cover vegetation (%) (min-max)
2000	0	3	0	0	2	46.3 (53–32)	80 (55–95)
2100	1	3	1	0	2	45.7 (56–34)	80 (75–85)
2200	0	3	1	0	3	56 (58–54)	82 (60–95)
2300	0	3	0	0	2	39 (49–36)	78 (55–98)
2400	1	2	1	0	2	39.3 (45–36)	83 (70–95)
2500	0	3	0	0	3	40.7 (44–39)	63 (60–65)
2600	1	2	1	0	2	38.7 (44–35)	86 (70–98)
2700	2	2	0	0	2	38 (45–27)	62 (55–70)
2800	0	3	0	0	2	35 (43–26)	67 (40–85)
2900	1	3	0	0	2	44.7 (56–38)	90 (80–95)
3000	2	3	0	0	2	37.7 (43–29)	67 (50–80)
3100	3	1	0	0	2	31.7 (39–28)	70 (60–80)
3200	3	1	0	0	2	30 (39–24)	67 (55–75)
3300	3	2	0	0	2	23.3 (24–23)	68 (55–80)
3400	3	1	1	0	2	27.7 (35–21)	75 (70–80)
3500	2	2	0	0	3	28.3 (35–23)	67 (60–75)
3600	2	2	1	0	2	27.3 (30–24)	60 (40–85)
3700	3	1	0	0	1	24.3 (30–19)	38 (35–45)
3800	3	2	0	0	2	19 (23–17)	53 (40–80)
3900	3	1	0	0	1	16.3 (21–14)	35 (35–35)
4000	2	2	0	0	2	14.7 (17–10)	37 (10–65)
4100	3	1	0	0	1	12 (13–12)	23 (15–35)
4200	3	1	1	1	1	7.3 (9–6)	18 (4–45)
4300	3	1	0	0	1	8 (12–4)	5 (2–8)
4400	3	0	0	0	0	5 (6–4)	3 (3–3)
4500	1	0	0	0	0	5	5
Total	48	48	7	1	46		

At each elevation, three plots were considered, thus the maximum possible number of times a model can be recorded as a best model is three, except for the last elevation (4500 m), for which only one plot was used because of the limited area available at the peak. Mean of species richness was calculated as average number of species per plot. Total cover vegetation within each plot was visually estimated during field data sampling.

**Fig. 2:**
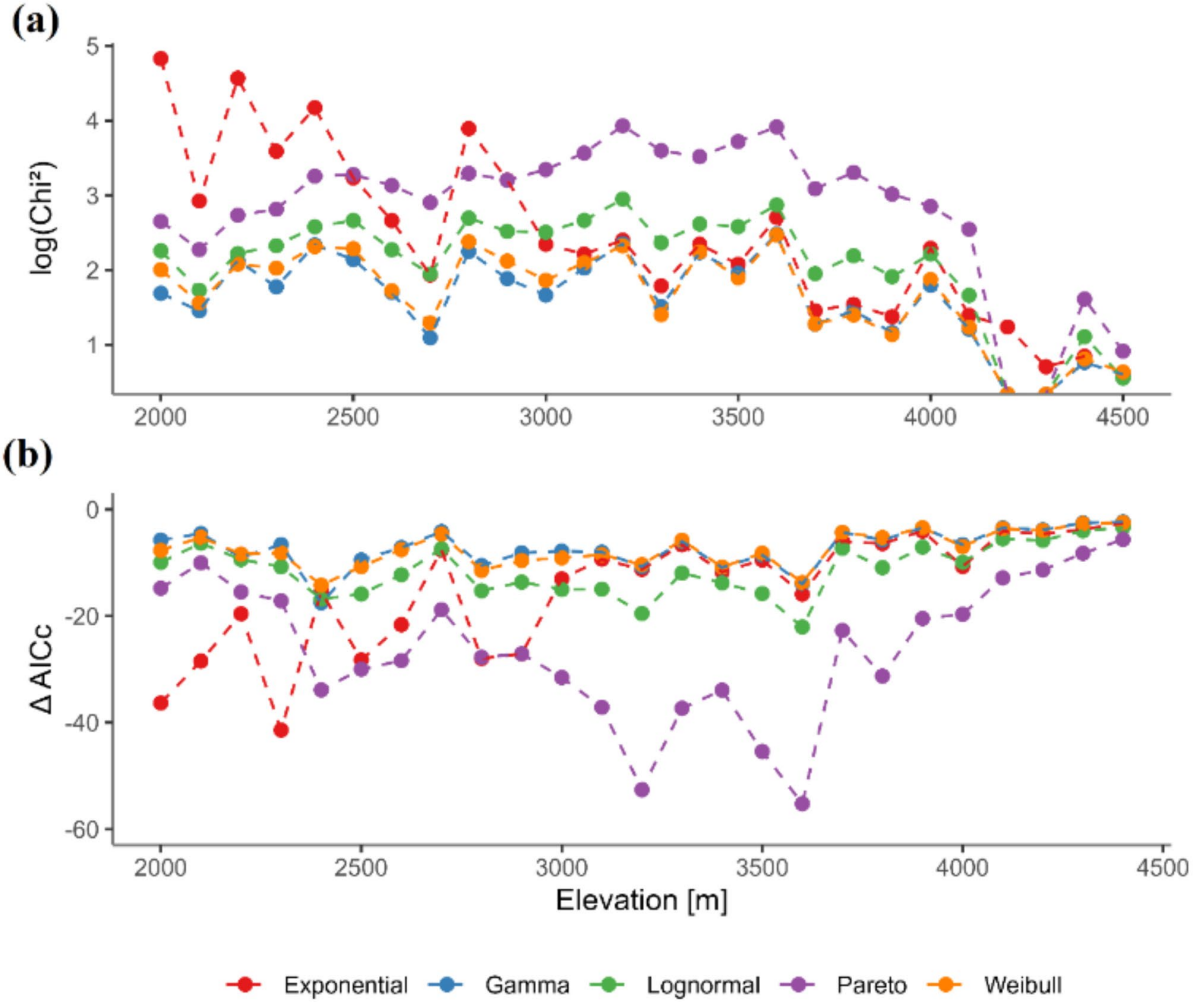
Model fit evaluation for plant species abundance distributions (SADs) along an elevational gradient in the Alborz Mountains (Iran). (a) Log-transformed χ^2^-test values for different parametric models (exponential, gamma, lognormal, Pareto, and Weibull), representing the goodness-of-fit between observed species richness in each cover class and expected values from the models. Lower Chi² values indicates a better fit. (b) ΔAICc values for the same five models, where lower values indicate a better fit. Three data points from the exponential model were removed for clarity as they had ΔAICc values below − 100.

**Fig. 3:**
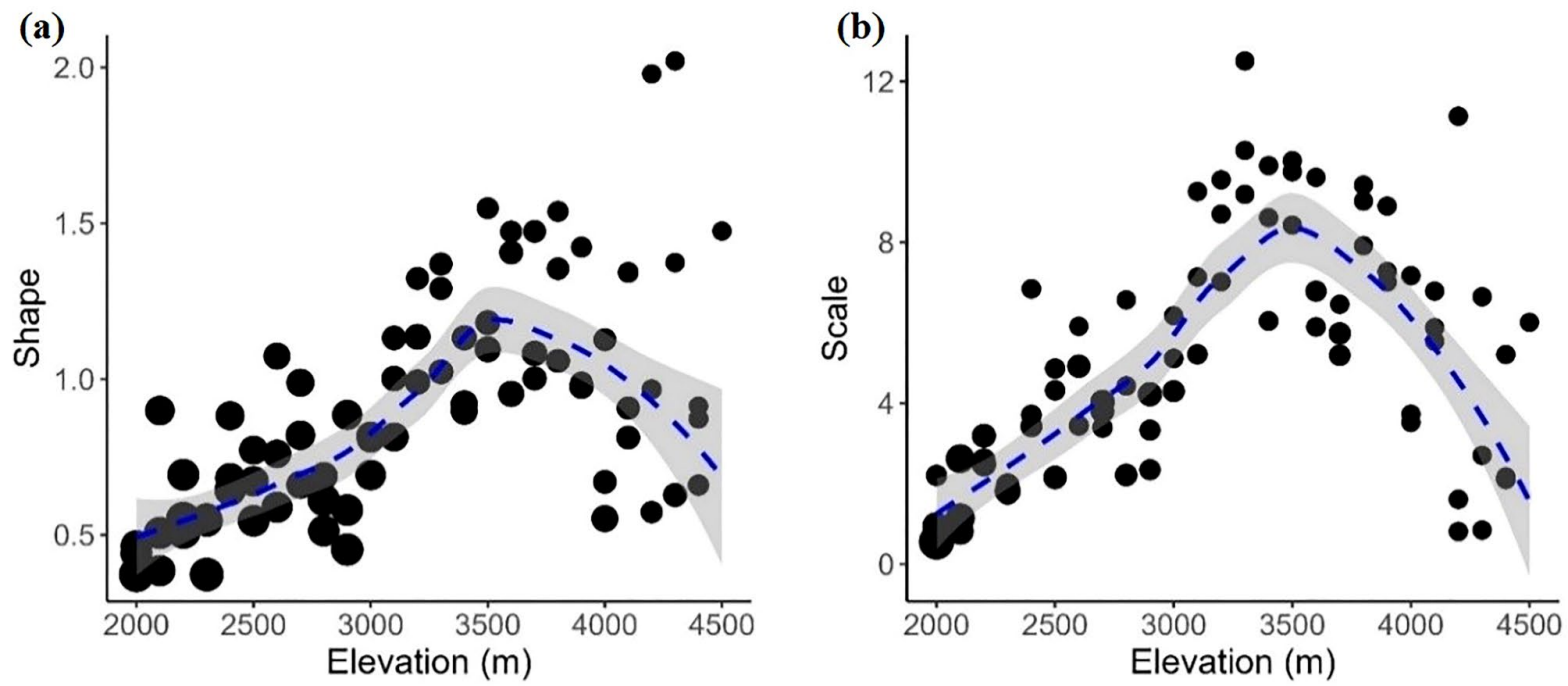
Changes of Weibull parameters for vascular plant species abundance distributions (SADs) along a transect in the Alborz Mountains (Iran): (a) relationship between φ (shape) and elevation; (b) relationship between λ (scale) and elevation. Dashed lines represent the loess smoother. Grey areas show the standard error of the smoother. Dot size is proportional to the weighted regression by the inverse of the standard errors of the estimations.

Our fits to continuous probability functions showed that the exponential, gamma and Weibull models best described the SADs in most cases, whereas the lognormal and Pareto models were, in general, less adequate (Tables S1 and S2, Fig. S1). Both the exponential and the gamma distributions were recovered as plausible models in about 63% of cases, and the Weibull distribution in about 61% of cases. The lognormal distribution was among the plausible models in about 9% of cases, and the Pareto distribution in about 1%. In fact, although χ^2^ tests showed that many SADs did not deviate significantly from the lognormal and the Pareto models (Fig. 2a, Table S2), other models were best supported by the data on the basis of the AICc values (Table S1). From 2,000 m to 3,000 m, the gamma and Weibull models were almost always the only plausible models. Above 3,000 m, the exponential model was best supported by the observed SADs (Fig. 2b). Overall, although the Weibull model was not always the most frequently selected model in each elevational band, it was well supported along the entire elevational gradient (Fig. 2; Table 1, Table S1). The two parameters φ and λ of the Weibull function peaked just above the centre of the investigated elevational gradient (ca. 3,200-3,800 m), whereas their lower values were observed at the extremes (Fig. 3).

## Discussion

Empirical studies on SADs in plant communities using parametric modelling were restricted to trees due to their countable nature^32–34^. However, for most plant species (but also for some animals like corals) using counts as a measure of abundance may be impractical or theoretically inappropriate, and cover measures are commonly used to express relative abundance. Oddly enough, some of the many SAD models commonly fitted to count data are indeed continuous probabilities distributions, like all the distributions used in this work. We thus provided the generic formulation and a computational implementation to fit these distributions to cover data, and applied these continuous models to investigate changes in SAD parameters in plant communities along an elevational gradient.

As is common in plant community research, we used cover values recorded using a predefined class-based scale as our starting point. Among the various methods for assessing plant relative abundance in the field, we opted for the Londo scale, as it contains a relatively large number of classes, i.e. a total of thirteen cover classes are possible^35^. This offered the advantage to define a large number of bins using the endpoints of Londo classes (if classes are too few, and hence to large, many values will fall in the same bin, and this will obscure the true SAD shapes). However, the approach described in this paper can be applied to any other recording scale (e.g., the Braun-Blanquet scale). Testing the sensitivity of the estimates to different cover scales is an interesting path for further studies. As our approach uses continuous distributions, it can be used also for biomass data, although they are less frequently applied as measures of abundance because recording biomass is more complex^3,15^. Point quadrat cover or density might be even better measures of abundance than cover, but they are not so commonly used in plant communities as cover classes.

By applying different types of probability distributions to cover data of samples of plant communities, we could interpret changes in parameters describing SADs along an elevational gradient. We found that both the gamma and Weibull distributions adequately described the SADs in most cases, which can be explained by their flexibility. The gamma distribution is known to have the capacity to accommodate shapes close to the lognormal SADs typical of species rich assemblages, as well as unimodal and right skewed distributions^4,36^, and the Weibull model is known to be able to fit the most commonly observed SAD shapes^17,33^. However, for higher-elevation plots, the exponential distribution emerged as the best model, despite the fact that gamma and Weibull models encompass the exponential as a special case. This can be explained by the behaviour of these functions in terms of AIC values. The flexibility of gamma and Weibull models comes at the cost of an additional shape parameter with respect to the exponential one: thus, these two distributions incur in a heavy penalty in the calculation of the AIC, when the version corrected for small sample sizes is used. For instance, for a sample size of 10 species, an extra parameter increases the ΔAICc by 3.21 units, while with four species, the penalty raises in 12 units, compared to a fixed penalty of 2 units in uncorrected AIC (Table S3). Consequently, the single-parameter exponential distribution proved to be the most parsimonious for species-poor SADs at higher elevations, as Weibull and Gamma distributions risked overfitting.

We found that both parameters of the Weibull distribution (i.e. the shape φ and scale λ) showed unimodal patterns along the gradient. Low values of φ and λ indicate an excess of abundant species and a low variability, respectively^33^. Low φ and λ values at the extremes of the investigated gradient therefore suggest that the communities occupying the lowest and highest parts were both largely dominated by few common species of similar abundances. In contrast, the centre of the gradient was occupied by communities with high variation in species abundances, in which many species are rare but some are abundant, which indicates that communities in the middle of the gradient were more “diverse”^33^. This pattern supports our hypothesis about how the interplay between biotic interactions and environmental filtering influences SAD shapes. In the lowest part of our study gradient the importance of biotic interactions is higher than that of abiotic factors, leading to rich communities that are dominated by some species. Previous research in the same study system revealed that, because of increasingly harsher conditions with increasing elevation, communities are more profoundly shaped by environmental filtering^37^. This reduces the signature of competition, because only the few species that can withstand the stressful conditions that characterise these environments endure in smaller spots, thus producing communities with less pronounced differences in species cover. Our previous research on plant functional traits in the same study area^38^ further supports this interpretation, showing that while lower elevation areas contain a variety of plant functional types (PFTs), higher elevations host fewer PFTs, with the highest elevations (above 4,000 m) containing only a single dominant PFT - tiny rosette plants with soft mesomorphic leaves (e.g., *Galium aucheri* and *Veronica aucheri*). This pattern highlights the role of environmental filtering, rather than biotic interactions in shaping community structure in Mt. Rostam-Nisht, with only species showing traits adapted to extreme climatic conditions and high rock cover being able to persist at higher elevations. At intermediate elevations, we observed a mix of functional types, including tall herbs (e.g., *Prangos uloptera*) and cushion plants (e.g., *Acantholimon hohenackeri* and *Astragalus macrosemius*), suggesting a balance between adaptive and competitive interactions. In addition, our findings indicate that plant traits such as growth forms are strongly associated with elevation further emphasizing that species distribution along the elevational gradient is primarily determined by adaptation to environmental conditions rather than competition. Finally, we observed that at the highest elevations (i.e., above 3,900 m), environmental conditions are so harsh that even the most successful species have low abundances. More details about the functional adaptations of indicator species and PFTs along the investigated elevational gradient and their relationships with environmental conditions are given in previous studies ^37–39^

Surprisingly, the lognormal distribution (which is commonly referred to represent species rich communities that are well balanced in terms of rare and common species^40^) was poorly supported along the elevational gradient. This may be the reflection of an excess of rare species, compared to that expected by the lognormal distribution, especially at intermediate elevations. This situation was first addressed by the introduction of the log-series distribution, which was deduced as a special discrete case of the gamma distribution^41^ as a result of stochastic birth-death-immigration processes^42,43^. Other models used the log-series to advance the idea that the number of rare species increases with immigration from a large species pool^44^. Among these ideas, the insight that communities are composed by core and transient species^32,45^ provides a reasonable explanation for the excess of rare species that we found in many plots as the consequence of populations that endure under suboptimal conditions for a while in these sites. This may be particularly true for the communities of the central part of our gradient. The maintenance of rare species by immigration of transient species may occur at all elevations, but the importance of this influx of transients (which will increase the number of rare species) is expected to be prominent in the central part of the gradient, because: (a) the intermediate environmental conditions that characterise the central part of the gradient may represent suboptimal but still viable conditions for more species with higher degrees of adaptation to the extremes of the elevation ranges, and (b) intermediate elevations are close to both the lower and to the higher areas, from which they can receive transients. While we highlight the potential role of transient species in shaping SADs, particularly at intermediate elevations, further research is needed to comprehensively explore their influence. Incorporating long-term datasets or more sophisticated models (e.g., spatially explicit meta-community models and stochastic simulation models) could provide insights into the effects of immigration and temporal variability on SADs, helping to elucidate the complex interactions that shape SADs across varying ecological landscapes. According to this hypothesis, the SAD is expected to show its mode in an intermediate abundance class rather than in the lowest abundance class^45^. For the Weibull distribution, such feature occurs if the shape parameter is larger than one. Indeed, the largest values of the estimated shape parameters were concentrated in the central part of the studied gradient. This explanation could be further explored and tested in the future by an analysis of temporal changes in species composition. If the SADs are strongly influenced by transient species, we would expect a larger temporal beta-diversity in these intermediate elevation areas. We acknowledge that our study focuses on a single vegetation component (herbaceous plants) within one ecological region (the Alborz Mountains), which limits the generalisability of our results. For example, herbaceous plants in tropical rainforests may exhibit different patterns of SAD due to differences in species composition, environmental factors and ecological interactions. However, the methods used in this study can be broadly applied and adapted for research in other regions and ecosystems, thus providing wide opportunities for comparative analyses. In particular, further research at different elevational gradients and in a wider range of ecological regions is therefore needed to validate and refine our findings. This could include, for example, studies in the lowlands of the Andes or the Alpine regions of Europe, where different climatic conditions and species composition may lead to contrasting patterns of SAD. In particular, it would be valuable to examine gradients starting at lower elevations or located in different ecoregions to test the generalisability of our conclusions. The effects of elevation on SADs in high elevation mountain ecosystems, such as the Himalayas or the Andes, could provide additional insights into the role of environmental filtering and species turnover at higher elevations.

Interestingly, the vegetation of the Alborz Mountains has some similarities with other temperate mountain regions, such as the Caucasus, the Pyrenees or parts of the Alps, where similar elevational gradients and herbaceous plant communities can be found^46,47^. Comparisons between these regions could therefore help us to understand whether the patterns observed in the Alborz Mountains apply to these ecosystems.

It is also important to note that, as typical of elevational gradients^48^, vegetation cover in the study system tended to decline with increasing elevation^40^. Future research would be useful to investigate the impact of variation of total cover in the SAD shapes. Widening the scope of such research would provide a more comprehensive understanding of SAD patterns and their underlying mechanisms across ecosystems. Finally, our research was affected by uncertainties about the exact coverage value of each species due to the use of cover classes instead of direct accurate estimates. When feasible, adoption of field protocols to estimate plant species cover directly in the field without the use of classes would be preferable in future work, especially in areas with small number of species. By paving the way to study cover data through continuous probability distributions, we encourage researchers to further investigate SAD patterns of plant communities to understand how they change across ecosystems.

## Conclusion

In this study, we have demonstrated that models traditionally used for fitting SAD patterns using frequency distributions of individuals can effectively be adapted for continuous data like cover proportions. This adaptation has allowed us to explore variations in SAD shapes across grassland communities along an elevational gradient. Since plant communities are commonly described using cover class scales, we demonstrated how modelling SADs by fitting probability distributions to cover data with the maximum likelihood method offers an alternative to traditional approaches based on rank-abundance plots. Using a case study of plant communities distributed along a wide elevational gradient, we investigated how SADs change along the gradient. Specifically, we found that the gamma and Weibull distributions provided the best fit in most communities along the elevational gradient possibly in response to increasing harsher conditions, changes in biotic interactions, and immigration patterns. Our results pave the way for the use of cover data in SAD modelling and advocate for further research on elevational gradients as privileged contexts to investigate SAD patterns and the underlaying mechanisms. Additionally, the integration of multiscale environmental data and the exploration of other biogeographical settings could further validate the generalisability of our findings and refine our theoretical models of species distribution and abundance.

## Materials and methods

### Data collection

We used data collected from plots located along an elevational gradient in the central part of the Alborz Mountains (Iran). The Alborz range includes several peaks surpassing 4,000 m elevation (Mt. Damavand, 5,671 m a.s.l., is the highest peak in Iran). This mountain range is largely occupied by the Caspian Hyrcanian mixed forests (a type of deciduous broadleaved forests, recognized as a UNESCO World Heritage Site since 2019) up to about 2,800 m, while at higher elevations grasslands and steppes succeed^49^. Hemicryptophytes are the prevailing life forms, but thorn cushions of chamaephytes are common in sub-alpine zones with low temperatures and long persistence of the snow cover^50^.

With increasing elevation, not only climate becomes harsher due to the decrease in temperature, but also substrate characteristics change: soils become thinner, and the proportion of rocky surfaces increases, which progressively restricts plant cover to scree vegetation^37^. High elevations are colonised by only few species^38^.

**Fig. 4:**
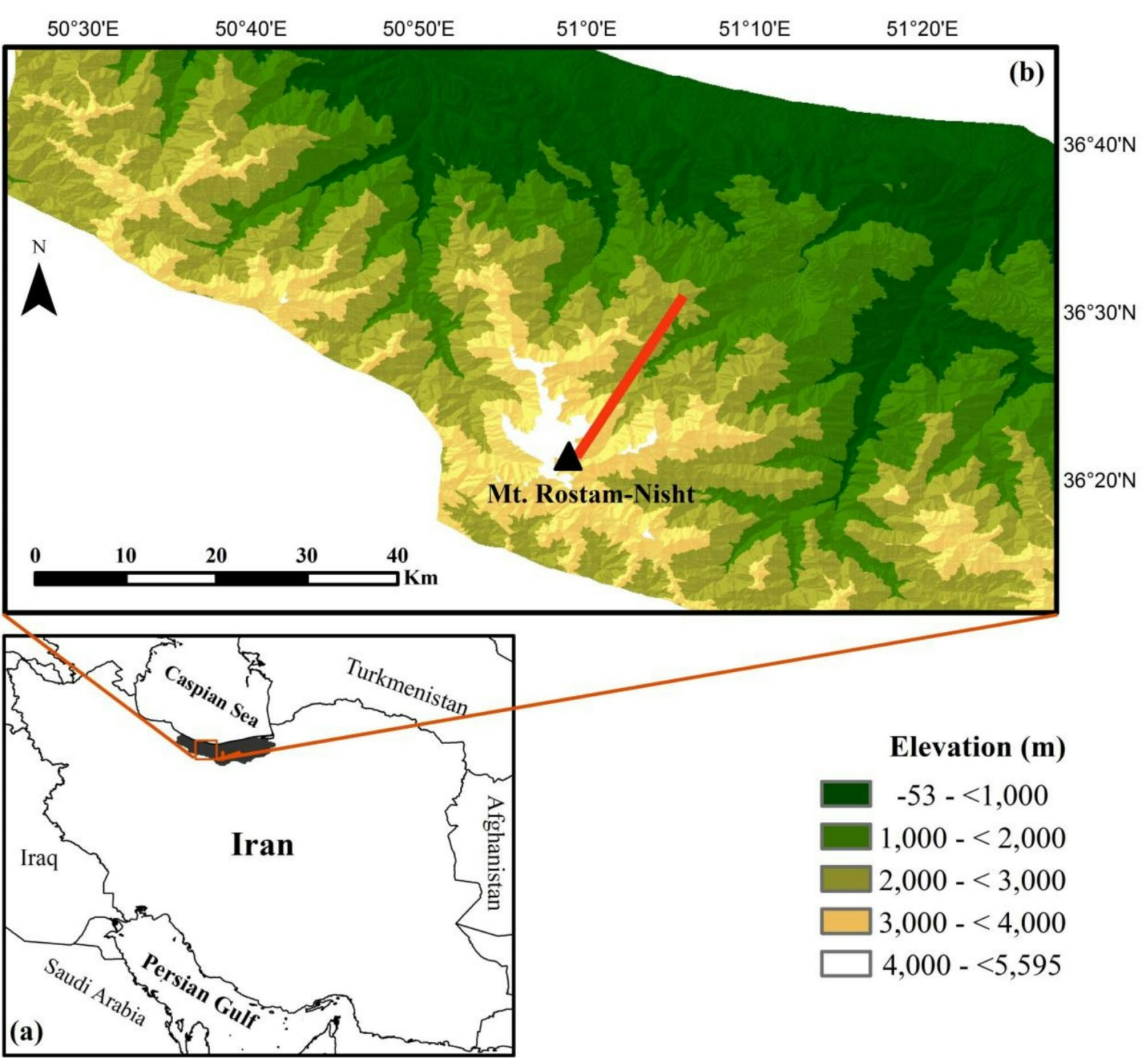
Map of the study area. (a) Iran and surrounding countries. The rectangle shows the location of the study area in the central Alborz Mountains. Country boundaries were derived from GADM (Global Administrative Areas; https://gadm.org/index.html). (b) Location of the elevational gradient (red solid line) studied on Mt. Rostam-Nisht. Elevation data were sourced from NASA SRTM (Shuttle Radar Topography Mission; https://www.earthdata.nasa.gov/data/instruments/srtm).

The elevational gradient investigated in the present study started just above the treeline on Mt. Rostam-Nisht (N 36° 26′ 16.1″, E 051° 03′ 23.2″ and N 36° 24′ 05.9″, E 050° 57′ 43.7″) and extended from 2,000 to 4,500 m (Fig. 4). This gradient was divided into 100 m elevational belts, and three plots of 100 m^2^ (i.e. quadrats of 10 m × 10 m) were randomly allocated in each belt for a total of 76 vegetation plots. Data collection was performed during the summer of 2014 by the first author, who also identified plant species based on *Flora Iranica*^51^ and *Flora of Iran*^52^. Efforts were made to select, within each elevational belt, plots with similar environmental conditions, such as slope and aspect, but which were representative of the main conditions of the belt (for example, we avoided placing plots in valley bottoms). We ensured that areas subject to grazing were avoided. Plots were also carefully placed to minimise spatial autocorrelation by avoiding plots that were too close together.

Plant species cover in each plot was recorded using the cover classes of the Londo scale^35^. The Londo scale uses thirteen cover classes that express the actual coverage values quite finely^35^. After recording species cover using Londo classes in the field, these were transformed into proportions prior analysis using, as best estimate of individual species cover, the midpoints of the class to which a species’ cover was assigned. Although this method may appear convoluted, and implies a certain loss of information compared with the possibility of recording directly the proportional cover in the field using a continuous scale instead of classes, it was used because plant ecologists usually do not estimate plant cover using continuous scales, but adopt protocols that refer to cover classes, of which the Londo scale is just an example. Another, widely applied scale, is for example the Braun-Blanquet scale, whose classes are then commonly transformed into percentages for analyses^53,54^. Thus, the procedure illustrated here can be equally applied to most datasets available in plant ecology.

### Statistical analyses

We applied the following continuous distribution models to fit SADs using plant cover as measure of species abundance: the exponential, gamma, lognormal, Pareto and Weibull. These models are among the most commonly used distributions in parametric modelling of SADs^33,36,55^. Models were fitted to the frequency of species in each cover class by finding the values of the parameters of each distribution model that maximise the log-likelihood function:

$$\mathcal{L}\left( \theta \right)=\mathop \sum \limits^{C} {n_i}{\text{ln}}\left( {{P_i}} \right)$$

where *C* is the total number of cover classes, *n*_*i*_ is the number of species recorded in the *i-th* cover class, and *P*_*i*_ is the probability attributed by the distribution model to the observation of one species in class *i*, which depends on the vector *θ* of free parameters of the distribution model:

$${P_i}=\mathop \smallint \limits_{{{L_i}}}^{{{U_i}}} F\left( {x|\theta } \right)dx$$

where *F*(*x*|*θ*) is the value of the probability density function for a cover value *x* under parameter values fixed at *θ*, and *U*_*i*_ and *L*_*i*_ are the lower and upper limits of the cover class *i*. The maximum likelihood estimates were found by numerical optimisation with the newly introduced functions *fitexpC*,* fitgammaC*, *fitlnormC*, *fitparetoC*, and *fitweibullC* in the “sads” package^56^ within the statistical software environment R 4.2.2 ^57^. We used the Londo scale breakpoints, (i.e., 0, 1, 3, 5, 10, 15, 25, …, 95, 100) to identify classes, that is, the limits *U*_*i*_ and *L*_*i*_, as defined above.

To identify the model(s) with the best predictive accuracy for the sampled SADs, we used Akaike’s information criterion corrected for small sample sizes (AICc) and selected the model(s) with the lowest AICc as the best supported by the data^58^. Models with a ΔAICc ≤ 2 were considered as equally plausible models. We also evaluated the goodness-of-fit of the models using χ^2^ tests (with *p*-values above 5% indicating adequate fit), to avoid comparisons between models from which the observed distributions deviated significantly from the expected ones.

Since the Weibull distribution was among the models that adequately described SADs in most cases, and its parameters are ecologically meaningful^17,33^, we further analysed how Weibull parameters varied along the elevational gradient. The two parameters of the Weibull distribution are the shape parameter φ, which expresses excess of either highly abundant species (low φ) or rare species (high φ), and the scale parameter λ, which expresses variability in species abundances^59^. Finally, we plotted the estimates of shape and scale and the observed species richness for each plot against elevation and used a loess-smoother to display the trend in the data using the *ggplot* function of the R package “ggplot2”^60^.

## Electronic supplementary material

Below is the link to the electronic supplementary material.

Supplementary Material 1

Supplementary Material 2

## Data Availability

The datasets generated during the current study are available in the Supplementary Material 2.
