# Supplementary material for: Use of cover data to model species abundance distributions through continuous probability functions
Source: Sci Rep. 2025 Apr 2;15:11361. doi: 10.1038/s41598-025-94587-w (PMC11965384; doi:10.1038/s41598-025-94587-w)

**Supplemental Information for:**

# **Use of cover data to model Species Abundance Distributions (SADs) through continuous probability functions**

Halime Moradi, Paulo Inácio Prado, Jens Oldeland, Paulo A. V. Borges, Simone Fattorini

# **Table S1**- ΔAICc values for different models used to fit plant species abundance distributions along an elevational gradient in the Alborz Mountains (Iran). ΔAICc≤ 2 are in bold. s**hape (φ)** and **scale (λ)** **parameters** of the Weibull distribution model.

| Elevation (m) | Exponential | Gamma | Lognormal | Pareto | Weibull | Shape parameter (φ) | Scale parameter (λ) |
| --- | --- | --- | --- | --- | --- | --- | --- |
| 2000 | 20.47 | **0.00** | 4.33 | 8.57 | **1.97** | 0.55 | 2.49 |
| 2000 | 24.20 | **0.00** | 2.15 | 4.62 | **0.90** | 0.51 | 2.59 |
| 2000 | 48.30 | **0.00** | 4.97 | 9.07 | 2.64 | 0.69 | 3.19 |
| 2100 | **0.00** | **1.53** | 2.25 | 8.26 | **1.45** | 0.90 | 2.62 |
| 2100 | 19.59 | **0.00** | **0.97** | 3.11 | **0.17** | 0.51 | 1.15 |
| 2100 | 24.01 | **0.00** | 3.85 | 6.76 | 2.10 | 0.39 | 0.82 |
| 2200 | 26.57 | **1.64** | **0.08** | 4.89 | **0.00** | 0.47 | 2.21 |
| 2200 | 31.46 | **0.00** | 3.31 | 10.08 | **0.74** | 0.44 | 0.56 |
| 2200 | 6.89 | **0.00** | 2.60 | 12.21 | **0.10** | 0.37 | 0.95 |
| 2300 | 37.44 | **0.00** | 6.36 | 11.42 | 3.40 | 0.37 | 1.74 |
| 2300 | 10.51 | **0.00** | 3.61 | 8.41 | **1.27** | 0.55 | 1.96 |
| 2300 | 17.59 | **0.00** | 4.67 | 10.90 | **1.63** | 0.55 | 1.82 |
| 2400 | 8.95 | **0.84** | **1.51** | 10.19 | **0.00** | 0.64 | 3.71 |
| 2400 | 5.08 | **0.00** | 3.88 | 11.50 | **0.82** | 0.68 | 3.43 |
| 2400 | **0.00** | **0.33** | 11.15 | 34.05 | **1.35** | 0.88 | 6.85 |
| 2500 | 2.06 | **0.00** | 7.34 | 20.10 | **1.37** | 0.77 | 4.86 |
| 2500 | 7.26 | **0.00** | 7.49 | 18.52 | **1.99** | 0.67 | 4.32 |
| 2500 | 15.20 | **0.00** | 2.33 | 7.45 | **0.49** | 0.55 | 2.16 |
| 2600 | **0.00** | 2.16 | 8.80 | 28.62 | **1.96** | 1.07 | 4.92 |
| 2600 | 2.79 | **0.00** | 10.35 | 26.55 | 2.06 | 0.76 | 5.91 |
| 2600 | 10.81 | **0.00** | **2.00** | 7.08 | **0.31** | 0.59 | 3.45 |
| 2700 | **0.43** | **0.00** | 4.59 | 16.10 | **0.50** | 0.82 | 3.80 |
| 2700 | 3.96 | **0.00** | 4.17 | 10.46 | **1.11** | 0.66 | 3.40 |
| 2700 | **0.00** | 2.20 | 5.06 | 19.81 | 2.20 | 0.99 | 4.03 |
| 2800 | 12.96 | **0.00** | 4.33 | 13.72 | **0.75** | 0.61 | 4.43 |
| 2800 | 3.35 | **0.00** | 6.43 | 16.25 | **1.58** | 0.69 | 6.57 |
| 2800 | 17.06 | **0.00** | 5.23 | 11.24 | 2.05 | 0.51 | 2.22 |
| 2900 | 25.53 | **0.00** | 8.09 | 15.30 | 3.82 | 0.45 | 2.35 |
| 2900 | 13.86 | **0.00** | 4.98 | 12.54 | **1.54** | 0.58 | 3.34 |
| 2900 | **0.00** | **0.69** | 8.16 | 28.98 | **1.15** | 0.89 | 4.23 |
| 3000 | **0.00** | **0.08** | 4.47 | 15.18 | **0.62** | 0.81 | 6.18 |
| 3000 | 6.60 | **0.00** | 8.60 | 20.88 | 2.22 | 0.69 | 5.12 |
| 3000 | **0.69** | **0.00** | 6.97 | 20.37 | **1.02** | 0.82 | 4.30 |
| 3100 | **0.66** | **0.00** | 6.70 | 21.18 | **0.91** | 0.81 | 5.22 |
| 3100 | **0.00** | 2.15 | 9.39 | 25.98 | 2.33 | 1.00 | 7.14 |
| 3100 | **0.00** | 2.32 | 11.60 | 33.06 | **1.86** | 1.13 | 9.26 |
| 3200 | **0.00** | 2.12 | 12.32 | 40.50 | **1.54** | 1.14 | 8.70 |
| 3200 | **0.00** | **1.63** | 9.01 | 29.57 | **0.41** | 1.32 | 9.56 |
| 3200 | **0.00** | 2.08 | 9.39 | 24.58 | 2.34 | 0.99 | 7.02 |
| 3300 | **0.00** | **1.83** | 9.47 | 30.23 | **0.73** | 1.29 | 10.29 |
| 3300 | **0.00** | 2.37 | 7.25 | 19.11 | 2.39 | 1.02 | 9.19 |
| 3300 | **0.42** | **0.50** | 4.17 | 28.46 | **0.00** | 1.37 | 12.51 |
| 3400 | **0.00** | 2.06 | 6.64 | 24.65 | **1.81** | 1.13 | 8.61 |
| 3400 | **0.00** | 2.24 | **1.54** | 13.56 | 2.03 | 0.90 | 9.91 |
| 3400 | **0.00** | **1.33** | 9.80 | 27.26 | **1.99** | 0.92 | 6.05 |
| 3500 | **0.00** | 2.25 | 13.21 | 40.29 | **1.92** | 1.10 | 9.76 |
| 3500 | 2.22 | **1.74** | 8.16 | 33.21 | **0.00** | 1.55 | 10.03 |
| 3500 | **0.00** | **1.61** | 4.55 | 20.36 | **1.39** | 1.18 | 8.43 |
| 3600 | **0.00** | **1.79** | 8.58 | 21.09 | 2.23 | 0.95 | 5.90 |
| 3600 | **1.19** | **2.00** | 10.29 | 33.89 | **0.00** | 1.47 | 9.62 |
| 3600 | 2.10 | **0.00** | **0.60** | 18.10 | **0.31** | 1.41 | 6.79 |
| 3700 | **0.00** | 2.45 | 6.52 | 17.01 | 2.51 | 1.00 | 6.46 |
| 3700 | **0.00** | 2.24 | 6.87 | 21.60 | 2.08 | 1.08 | 5.20 |
| 3700 | **1.92** | **0.15** | 2.27 | 20.36 | **0.00** | 1.47 | 5.74 |
| 3800 | **0.00** | **1.52** | 8.70 | 28.45 | **0.22** | 1.35 | 9.03 |
| 3800 | **0.78** | **1.09** | 5.58 | 23.75 | **0.00** | 1.54 | 7.92 |
| 3800 | **0.00** | 2.57 | 5.34 | 16.80 | 2.52 | 1.06 | 9.42 |
| 3900 | **0.00** | 2.62 | 6.28 | 13.63 | 2.76 | 0.99 | 7.01 |
| 3900 | **0.00** | **1.24** | 3.88 | 16.42 | **0.75** | 1.42 | 8.90 |
| 3900 | **0.00** | 2.29 | 6.76 | 17.73 | 2.44 | 0.98 | 7.27 |
| 4000 | **0.00** | **0.96** | 2.78 | 5.55 | **1.43** | 0.67 | 3.73 |
| 4000 | 5.18 | **0.00** | 4.08 | 8.64 | **1.55** | 0.55 | 3.53 |
| 4000 | **0.00** | 2.54 | 6.55 | 18.03 | 2.32 | 1.13 | 7.17 |
| 4100 | **0.00** | 2.46 | 5.33 | 12.01 | 2.69 | 0.91 | 6.79 |
| 4100 | **0.00** | 2.03 | 3.84 | 7.30 | 2.30 | 0.81 | 5.54 |
| 4100 | **0.00** | **1.93** | 3.14 | 8.69 | **1.72** | 1.34 | 5.88 |
| 4200 | **0.00** | 2.02 | 2.98 | 3.89 | 2.40 | 0.57 | 1.61 |
| 4200 | **0.00** | **1.91** | **1.91** | **1.91** | **1.91** | 1.98 | 0.81 |
| 4200 | **0.00** | 4.87 | 6.82 | 10.33 | 4.99 | 0.97 | 11.14 |
| 4300 | **0.32** | **0.00** | 2.84 | 5.96 | **0.97** | 0.63 | 2.71 |
| 4300 | **0.00** | 3.18 | 4.83 | 10.80 | 2.85 | 1.37 | 6.65 |
| 4300 | **0.00** | 11.44 | 11.43 | 11.44 | 11.43 | 2.02 | 0.85 |
| 4400 | **0.00** | 11.89 | 12.67 | 14.03 | 11.96 | 0.91 | 5.21 |
| 4400 | **0.00** | 6.57 | 6.66 | 6.94 | 6.57 | 0.87 | 2.11 |
| 4400 | **0.00** | 3.79 | 4.72 | 5.74 | 4.08 | 0.66 | 2.17 |
| 4500 | **0.00** | 5.53 | 5.28 | 5.66 | 5.70 | 1.48 | 6.01 |

# **Table S2-** Chi-square tests for plant species abundance distributions along an elevational gradient in the Alborz Mountains (Iran). Chi-square tests were performed using the observed values of species richness in each cover class and the expected values obtained with different fitting distributions (exponential, gamma, lognormal, Pareto and Weibull).

|  | **Exponential** | | **Gamma** | | **Lognormal** | | **Pareto** | | **Weibull** | |
| --- | --- | --- | --- | --- | --- | --- | --- | --- | --- | --- |
| **Elevation (m)** | χ2 | p-value | χ2 | p-value | χ2 | p-value | χ2 | p-value | χ2 | p-value |
| 2000 | 36.32 | 0 | 7.92 | 0.791 | 13.79 | 0.315 | 21.1 | 0.049 | 10.47 | 0.575 |
| 2000 | 523.86 | 0 | 5.91 | 0.92 | 8.34 | 0.758 | 11.54 | 0.484 | 6.89 | 0.865 |
| 2000 | 103.17 | 0 | 3.4 | 0.992 | 7.62 | 0.814 | 11.73 | 0.468 | 5.68 | 0.932 |
| 2100 | 3.93 | 0.985 | 2.79 | 0.997 | 2.88 | 0.996 | 7.43 | 0.828 | 2.65 | 0.998 |
| 2100 | 44.17 | 0 | 4.46 | 0.974 | 6.43 | 0.893 | 9.22 | 0.684 | 5.14 | 0.953 |
| 2100 | 37.27 | 0 | 6.32 | 0.899 | 9.67 | 0.645 | 13.41 | 0.34 | 7.9 | 0.793 |
| 2200 | 296.69 | 0 | 13.1 | 0.362 | 10.50 | 0.572 | 13.45 | 0.337 | 11.04 | 0.525 |
| 2200 | 153.5 | 0 | 8.44 | 0.75 | 10.69 | 0.556 | 17.27 | 0.14 | 8.85 | 0.715 |
| 2200 | 19.58 | 0.075 | 5.37 | 0.944 | 7.00 | 0.858 | 15.75 | 0.203 | 5.24 | 0.949 |
| 2300 | 72.7 | 0 | 5.58 | 0.936 | 10.43 | 0.578 | 16.43 | 0.172 | 7.93 | 0.79 |
| 2300 | 22.88 | 0.029 | 10.84 | 0.543 | 14.92 | 0.246 | 22.44 | 0.033 | 11.96 | 0.449 |
| 2300 | 28.65 | 0.004 | 3.4 | 0.992 | 6.90 | 0.864 | 12.57 | 0.401 | 4.59 | 0.97 |
| 2400 | 1224.83 | 0 | 33.39 | 0.001 | 15.68 | 0.206 | 22.6 | 0.031 | 21.81 | 0.04 |
| 2400 | 11.55 | 0.482 | 1.92 | 1 | 4.90 | 0.961 | 11.48 | 0.488 | 2.61 | 0.998 |
| 2400 | 19.29 | 0.082 | 17.25 | 0.14 | 29.88 | 0.003 | 67.55 | 0 | 18.21 | 0.109 |
| 2500 | 19.53 | 0.077 | 15.89 | 0.197 | 25.97 | 0.011 | 49.44 | 0 | 17.34 | 0.137 |
| 2500 | 17.49 | 0.132 | 5.54 | 0.937 | 12.65 | 0.395 | 26.06 | 0.011 | 7.44 | 0.827 |
| 2500 | 47.71 | 0 | 7.01 | 0.857 | 9.00 | 0.703 | 14.46 | 0.272 | 7.39 | 0.831 |
| 2600 | 3.49 | 0.991 | 3.46 | 0.991 | 8.86 | 0.715 | 28.8 | 0.004 | 3.35 | 0.993 |
| 2600 | 20.86 | 0.052 | 14.81 | 0.252 | 23.47 | 0.024 | 47.51 | 0 | 16.01 | 0.191 |
| 2600 | 40.51 | 0 | 3.21 | 0.994 | 4.39 | 0.975 | 8.8 | 0.72 | 3.27 | 0.993 |
| 2700 | 3.97 | 0.984 | 0.83 | 1 | 4.39 | 0.975 | 14.6 | 0.264 | 1.24 | 1 |
| 2700 | 11.73 | 0.467 | 4.49 | 0.973 | 8.51 | 0.744 | 16.5 | 0.169 | 5.47 | 0.94 |
| 2700 | 7.14 | 0.848 | 7.14 | 0.848 | 9.23 | 0.683 | 25.37 | 0.013 | 7.13 | 0.849 |
| 2800 | 189.57 | 0 | 17.75 | 0.123 | 20.64 | 0.056 | 36.36 | 0 | 17.06 | 0.147 |
| 2800 | 15.34 | 0.224 | 6.82 | 0.87 | 12.79 | 0.385 | 26.75 | 0.008 | 8.2 | 0.769 |
| 2800 | 40.62 | 0 | 7.02 | 0.857 | 12.33 | 0.42 | 20.15 | 0.064 | 9.02 | 0.702 |
| 2900 | 43.51 | 0 | 9.61 | 0.65 | 16.67 | 0.163 | 26.88 | 0.008 | 12.41 | 0.413 |
| 2900 | 22.56 | 0.032 | 2.4 | 0.998 | 6.46 | 0.891 | 13.61 | 0.327 | 3.71 | 0.988 |
| 2900 | 15.27 | 0.227 | 12.4 | 0.414 | 17.76 | 0.123 | 40.78 | 0 | 12.59 | 0.399 |
| 3000 | 4.13 | 0.981 | 1.26 | 1 | 4.76 | 0.966 | 14.81 | 0.252 | 1.74 | 1 |
| 3000 | 22.94 | 0.028 | 13.45 | 0.338 | 24.41 | 0.018 | 46.94 | 0 | 15.81 | 0.2 |
| 3000 | 12 | 0.446 | 8.71 | 0.728 | 15.87 | 0.198 | 32.86 | 0.001 | 9.72 | 0.641 |
| 3100 | 9.28 | 0.679 | 5.58 | 0.936 | 10.83 | 0.543 | 25.29 | 0.014 | 6.33 | 0.898 |
| 3100 | 7.06 | 0.853 | 6.68 | 0.878 | 13.02 | 0.368 | 32.86 | 0.001 | 7.07 | 0.853 |
| 3100 | 11.7 | 0.47 | 11.75 | 0.466 | 21.03 | 0.05 | 53.23 | 0 | 12.13 | 0.435 |
| 3200 | 12.63 | 0.396 | 12.55 | 0.403 | 23.36 | 0.025 | 63.33 | 0 | 12.23 | 0.427 |
| 3200 | 12.92 | 0.375 | 11.7 | 0.47 | 20.94 | 0.051 | 59.2 | 0 | 10.63 | 0.561 |
| 3200 | 8.2 | 0.77 | 7.88 | 0.795 | 14.28 | 0.283 | 35.31 | 0 | 8.16 | 0.772 |
| 3300 | 8.21 | 0.769 | 7.89 | 0.793 | 15.91 | 0.195 | 46.85 | 0 | 7.58 | 0.817 |
| 3300 | 8.75 | 0.724 | 8.77 | 0.722 | 14.71 | 0.258 | 36.87 | 0 | 8.7 | 0.728 |
| 3300 | 2.98 | 0.996 | 1.35 | 1 | 5.18 | 0.952 | 28.21 | 0.005 | 1.02 | 1 |
| 3400 | 7.51 | 0.822 | 6.93 | 0.862 | 12.52 | 0.405 | 39.21 | 0 | 6.52 | 0.888 |
| 3400 | 20.22 | 0.063 | 19.8 | 0.071 | 16.08 | 0.187 | 30.46 | 0.002 | 19.25 | 0.083 |
| 3400 | 7.49 | 0.823 | 5.97 | 0.917 | 12.77 | 0.386 | 32.09 | 0.001 | 6.7 | 0.877 |
| 3500 | 14.12 | 0.293 | 14.12 | 0.293 | 24.87 | 0.015 | 63.69 | 0 | 14.41 | 0.275 |
| 3500 | 11.05 | 0.525 | 8.6 | 0.737 | 17.04 | 0.148 | 50.85 | 0 | 7.39 | 0.831 |
| 3500 | 3.26 | 0.993 | 2.91 | 0.996 | 5.45 | 0.941 | 21.7 | 0.041 | 2.79 | 0.997 |
| 3600 | 20.99 | 0.051 | 21.09 | 0.049 | 33.09 | 0.001 | 66.62 | 0 | 21.23 | 0.047 |
| 3600 | 17.81 | 0.122 | 15.77 | 0.202 | 26.89 | 0.008 | 73.14 | 0 | 14.63 | 0.262 |
| 3600 | 8.92 | 0.71 | 5.13 | 0.954 | 6.19 | 0.906 | 25.96 | 0.011 | 5.3 | 0.947 |
| 3700 | 2.81 | 0.997 | 2.66 | 0.998 | 5.87 | 0.923 | 16.69 | 0.162 | 2.82 | 0.997 |
| 3700 | 2.18 | 0.999 | 2.14 | 0.999 | 5.88 | 0.922 | 20.36 | 0.061 | 2.05 | 0.999 |
| 3700 | 12.92 | 0.375 | 8.09 | 0.778 | 10.13 | 0.605 | 31.09 | 0.002 | 8.04 | 0.625 |
| 3800 | 10.81 | 0.545 | 9.77 | 0.636 | 18.16 | 0.111 | 51.76 | 0 | 8.92 | 0.71 |
| 3800 | 7.1 | 0.851 | 6.03 | 0.915 | 11.04 | 0.526 | 28.16 | 0.005 | 5.56 | 0.901 |
| 3800 | 1.33 | 1 | 1.34 | 1 | 3.58 | 0.99 | 13.9 | 0.307 | 1.34 | 1 |
| 3900 | 5.3 | 0.947 | 5.21 | 0.951 | 9.57 | 0.653 | 22.7 | 0.03 | 5.3 | 0.947 |
| 3900 | 3.6 | 0.99 | 2.14 | 0.999 | 4.61 | 0.97 | 19 | 0.089 | 1.8 | 1 |
| 3900 | 3.26 | 0.993 | 2.95 | 0.996 | 7.02 | 0.857 | 19.71 | 0.073 | 3.19 | 0.994 |
| 4000 | 5.98 | 0.917 | 3.39 | 0.992 | 5.39 | 0.944 | 9.71 | 0.641 | 3.83 | 0.986 |
| 4000 | 16.16 | 0.184 | 6.62 | 0.881 | 9.93 | 0.622 | 16.26 | 0.18 | 7.6 | 0.816 |
| 4000 | 9.99 | 0.617 | 9.81 | 0.632 | 14.37 | 0.278 | 33.08 | 0.001 | 9.49 | 0.661 |
| 4100 | 4.02 | 0.983 | 3.47 | 0.991 | 5.76 | 0.928 | 13.13 | 0.359 | 3.69 | 0.988 |
| 4100 | 5.05 | 0.956 | 4.49 | 0.973 | 7.33 | 0.835 | 14.6 | 0.264 | 4.8 | 0.964 |
| 4100 | 3.23 | 0.994 | 2.41 | 0.998 | 3.47 | 0.991 | 10.82 | 0.545 | 2.3 | 0.999 |
| 4200 | 5.26 | 0.949 | 3.73 | 0.988 | 5.56 | 0.937 | 8.15 | 0.773 | 4.3 | 0.978 |
| 4200 | 1.05 | 0.994 | 0 | 1 | 0.00 | 1 | 0 | 1 | 0 | 1 |
| 4200 | 7.46 | 0.826 | 7.72 | 0.806 | 11.85 | 0.458 | 25.88 | 0.011 | 7.56 | 0.819 |
| 4300 | 7.98 | 0.787 | 5.17 | 0.952 | 7.84 | 0.798 | 12.9 | 0.376 | 5.75 | 0.928 |
| 4300 | 2.9 | 0.996 | 2.45 | 0.998 | 4.18 | 0.98 | 11.79 | 0.463 | 2.2 | 0.999 |
| 4300 | 0.36 | 1 | 0 | 1 | 0.00 | 1 | 0 | 1 | 0 | 1 |
| 4400 | 2.13 | 0.999 | 2.09 | 0.999 | 3.21 | 0.994 | 6.27 | 0.902 | 2.14 | 0.999 |
| 4400 | 1.41 | 1 | 1.34 | 1 | 1.65 | 1 | 2.47 | 0.998 | 1.36 | 1 |
| 4400 | 4.23 | 0.979 | 3.54 | 0.99 | 5.26 | 0.949 | 8.15 | 0.773 | 3.92 | 0.985 |
| 4500 | 2.18 | 0.999 | 1.83 | 1 | 1.74 | 1 | 2.5 | 0.996 | 1.89 | 0.997 |

# **Table S3-** Uncorrected ΔAIC values for different models used to fit plant species abundance distributions along an elevational gradient in the Alborz Mountains (Iran). Uncorrected ΔAIC ≤ 2 are in bold.

| Elevation (m) | Weibull | Gamma | Lognormal | Exponential | Pareto |
| --- | --- | --- | --- | --- | --- |
| 2000 | **1.97** | **0.00** | 4.33 | 20.76 | 8.57 |
| 2000 | **0.90** | **0.00** | 2.15 | 24.37 | 4.62 |
| 2000 | 2.64 | **0.00** | 4.97 | 48.47 | 9.07 |
| 2100 | **1.29** | **1.37** | 2.09 | **0.00** | 8.10 |
| 2100 | **0.17** | **0.00** | **0.97** | 19.78 | 3.11 |
| 2100 | 2.10 | **0.00** | 3.85 | 24.29 | 6.76 |
| 2200 | **0.00** | **1.64** | **0.08** | 26.73 | 4.89 |
| 2200 | **0.74** | **0.00** | 3.31 | 31.61 | 10.08 |
| 2200 | **0.10** | **0.00** | 2.60 | 7.06 | 12.21 |
| 2300 | 3.40 | **0.00** | 6.36 | 37.69 | 11.42 |
| 2300 | **1.27** | **0.00** | 3.61 | 10.79 | 8.41 |
| 2300 | **1.63** | **0.00** | 4.67 | 17.77 | 10.90 |
| 2400 | **0.00** | **0.84** | **1.51** | 9.19 | 10.19 |
| 2400 | **0.82** | **0.00** | 3.88 | 5.32 | 11.50 |
| 2400 | **1.16** | **0.13** | 10.96 | **0.00** | 33.86 |
| 2500 | **1.37** | **0.00** | 7.34 | 2.29 | 20.10 |
| 2500 | **1.99** | **0.00** | 7.49 | 7.47 | 18.52 |
| 2500 | **0.49** | **0.00** | 2.33 | 15.43 | 7.45 |
| 2600 | **1.75** | **1.95** | 8.59 | **0.00** | 28.42 |
| 2600 | 2.06 | **0.00** | 10.35 | 3.04 | 26.55 |
| 2600 | **0.31** | **0.00** | **2.00** | 11.08 | 7.08 |
| 2700 | **0.50** | **0.00** | 4.59 | **0.62** | 16.10 |
| 2700 | **1.11** | **0.00** | 4.17 | 4.30 | 10.46 |
| 2700 | **1.99** | **1.99** | 4.85 | **0.00** | 19.60 |
| 2800 | **0.75** | **0.00** | 4.33 | 13.17 | 13.72 |
| 2800 | **1.58** | **0.00** | 6.43 | 3.72 | 16.25 |
| 2800 | 2.05 | **0.00** | 5.23 | 17.31 | 11.24 |
| 2900 | 3.82 | **0.00** | 8.09 | 25.76 | 15.30 |
| 2900 | **1.54** | **0.00** | 4.98 | 14.08 | 12.54 |
| 2900 | **1.00** | **0.53** | 8.01 | **0.00** | 28.83 |
| 3000 | **0.54** | **0.00** | 4.39 | **0.24** | 15.11 |
| 3000 | 2.22 | **0.00** | 8.60 | 6.81 | 20.88 |
| 3000 | **1.02** | **0.00** | 6.97 | **0.91** | 20.37 |
| 3100 | **0.91** | **0.00** | 6.70 | **0.89** | 21.18 |
| 3100 | **2.00** | **1.83** | 9.07 | **0.00** | 25.65 |
| 3100 | **1.54** | **1.99** | 11.27 | **0.00** | 32.73 |
| 3200 | **1.31** | **1.88** | 12.08 | **0.00** | 40.26 |
| 3200 | **0.02** | **1.24** | 8.62 | **0.00** | 29.18 |
| 3200 | **2.00** | **1.74** | 9.05 | **0.00** | 24.24 |
| 3300 | **0.34** | **1.44** | 9.08 | **0.00** | 29.84 |
| 3300 | **1.98** | **1.96** | 6.85 | **0.00** | 18.70 |
| 3300 | **0.00** | **0.50** | 4.17 | **0.83** | 28.46 |
| 3400 | **1.47** | **1.72** | 6.30 | **0.00** | 24.31 |
| 3400 | **1.58** | **1.79** | **1.08** | **0.00** | 13.10 |
| 3400 | **1.73** | **1.07** | 9.55 | **0.00** | 27.01 |
| 3500 | **1.67** | **2.00** | 12.96 | **0.00** | 40.03 |
| 3500 | **0.00** | **1.74** | 8.16 | 2.63 | 33.21 |
| 3500 | **1.05** | **1.27** | 4.21 | **0.00** | 20.02 |
| 3600 | **1.92** | **1.49** | 8.28 | **0.00** | 20.79 |
| 3600 | **0.00** | **2.00** | 10.29 | **1.58** | 33.89 |
| 3600 | **0.31** | **0.00** | **0.60** | 2.42 | 18.10 |
| 3700 | **2.00** | **1.93** | 6.01 | **0.00** | 16.49 |
| 3700 | **1.78** | **1.94** | 6.57 | **0.00** | 21.30 |
| 3700 | **0.00** | **0.15** | 2.27 | 2.31 | 20.36 |
| 3800 | **0.00** | **1.30** | 8.48 | **0.19** | 28.23 |
| 3800 | **0.00** | **1.09** | 5.58 | **1.37** | 23.75 |
| 3800 | **1.93** | **1.98** | 4.75 | **0.00** | 16.21 |
| 3900 | **2.00** | **1.86** | 5.52 | **0.00** | 12.87 |
| 3900 | **0.00** | **0.49** | 3.13 | **0.01** | 15.67 |
| 3900 | **1.99** | **1.83** | 6.30 | **0.00** | 17.27 |
| 4000 | **0.47** | **0.00** | **1.82** | **0.25** | 4.59 |
| 4000 | **1.55** | **0.00** | 4.08 | 5.77 | 8.64 |
| 4000 | **1.73** | **1.95** | 5.96 | **0.00** | 17.44 |
| 4100 | **1.85** | **1.63** | 4.49 | **0.00** | 11.17 |
| 4100 | **1.37** | **1.10** | 2.90 | **0.00** | 6.36 |
| 4100 | **0.78** | **0.99** | 2.20 | **0.00** | 7.76 |
| 4200 | **0.38** | **0.00** | **0.96** | **0.18** | **1.87** |
| 4200 | **0.48** | **0.48** | **0.48** | **0.00** | **0.48** |
| 4200 | **1.99** | **1.87** | 3.82 | **0.00** | 7.33 |
| 4300 | **0.97** | **0.00** | 2.84 | **1.25** | 5.96 |
| 4300 | **1.12** | **1.45** | 3.10 | **0.00** | 9.07 |
| 4300 | **1.43** | **1.44** | **1.43** | **0.00** | **1.44** |
| 4400 | **1.96** | **1.89** | 2.67 | **0.00** | 4.03 |
| 4400 | **1.91** | **1.90** | **2.00** | **0.00** | 2.28 |
| 4400 | **1.08** | **0.79** | **1.72** | **0.00** | 2.74 |
| 4500 | **1.03** | **0.87** | **0.61** | **0.00** | **0.99** |

# **Figure S1**- Examples of histograms with abundance classes of plant species and fitted species abundance distributions (SADs) at different elevations in the Alborz Mountains (Iran). (a): Example of a SAD from 2300 m, where the gamma distribution performed well and the values of shape and scale of the Weibull distribution were low; (b): example of a SAD from 2500 m, where the Weibull distribution was the best fitted model and the values of shape and scale were high; (c): example of a SAD from 3700 m, where the exponential distribution was the best fitted model and values of shape and scale were high; (d): example of a SAD from 4400 m, where the exponential distribution was the best fitted model and values of shape and scale were low.


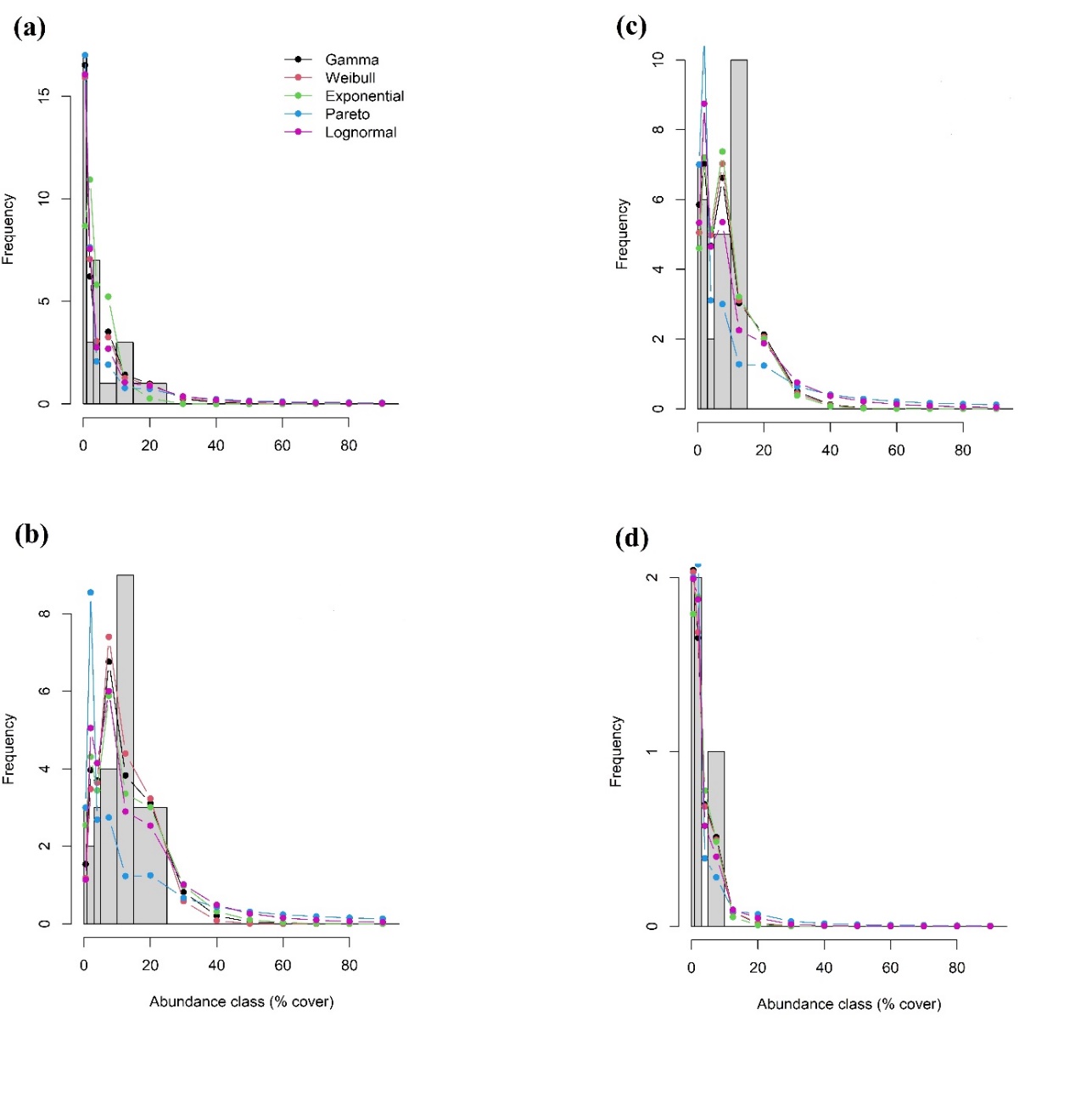

Supplement: Supplementary file 1 — Supplementary Material 1 [file 41598_2025_94587_MOESM1_ESM.docx]
